# Part 5: Allogeneic HSCT in refractory SJIA with lung disease; recent cases from centers in North America & Europe

**DOI:** 10.1186/s12969-023-00868-x

**Published:** 2024-01-05

**Authors:** Alexei A. Grom, Scott W. Canna, Rolla F. Abu-Arja, Rashmi Sinha, Luciana Peixoto, Elvira Cannizzaro, Shanmuganathan Chandrakasan, Kyla Driest, Rebecca Marsh, Bénédicte Neven, Karen Onel, Sampath Prahalad, Susan Prockop, Pierre Quartier, Johannes Roth, Grant Schulert, Juliana M.F. Silva, Donna Wall, Ulrike Zeilhofer

**Affiliations:** 1 Division of Rheumatology, Cincinnati Children’s Hospital Medical Center, University of Cincinnati, Cincinnati, OH USA; 2 Rheumatology & Immune Dysregulation, The Children’s Hospital of Philadelphia, Philadelphia, PA USA; 3 Department of BMT, Nationwide Children’s Hospital, Columbus, OH USA; 4 Systemic JIA Foundation, Cincinnati, OH USA; 5 Department of Rheumatology, University Children’s Hospital, Zurich, Switzerland; 6 Department of Pediatrics, School of Medicine, Emory University, Atlanta, GA USA; 7 Aflac Cancer and Blood Disorders Center, Children’s Healthcare of Atlanta, Emory University, Atlanta, GA USA; 8 Department of Rheumatology, Nationwide Children’s Hospital, Columbus, OH USA; 9 Division of Bone Marrow Transplantation and Immune Deficiency, Cincinnati Children’s Hospital Medical Center, Cincinnati, OH USA; 10 Pediatric Hematology-Immunology and Rheumatology Department, Necker-Enfants- Malades University Hospital, Paris, France; 11 Université Paris-Cité, Paris, France; 12 Department of Rheumatology, HSS, New York City, NY USA; 13 Dana Farber/Boston Childrens Hospital Center for Cancer and Blood Disorders, Boston, MA USA; 14 Kantonsspital Luzern, University of Luzern, Luzern, Switzerland; 15 Department of BMT, Great Ormond Street Hospital for Children, London, UK; 16 16. Blood and Marrow Transplant/Cellular Therapy, Division of Haematology/Oncology, Hospital for Sick Children Toronto, Toronto, Canada; 17 Department of BMT, University Children’s Hospital, Zurich, Switzerland

**Keywords:** Allogeneic HSCT, Refractory SJIA, SJIA-LD, MAS, HLA DRB1*15 alleles

## Abstract

It has been increasingly recognized that there is a subset of patients with refractory systemic JIA, who have failed all available medications and may benefit from HSCT. The increasing experience with HSCT in SJIA, suggests that despite the complicated post-HSCT course, short-term, the transplanted patients either achieved SJIA remission or reduced burden of disease. Longer follow-up, however, is needed to better define the long-term outcomes. The discussion at the NextGen 2022 conference was focused on the optimal timing for the procedure, the need for a good control of inflammatory SJIA activity prior to HSCT, and the role of the reduced intensity conditioning regimens as there was a remote concern that such regimens might increase the risk of SJIA relapse after the transplantation. There was unanimous agreement about the importance of long-term registries to address these questions.

## Introduction

In the last decade, the discovery that IL-1 & IL-6 are key therapeutic targets in systemic juvenile idiopathic arthritis (SJIA) and the introduction of IL-1 and IL-6 inhibiting biologics have led to a dramatic improvement in the outcome of typical features of this disease. At the same time, starting with the publication by Kimura in 2013, there has been a growing recognition of a subset of children with mostly systemic disease who are refractory to biologics and develop recurrent macrophage activation syndrome (MAS) as well as a seemingly new life-threatening pulmonary complication (SJIA-LD). These patients typically stay on multiple immunosuppressive medications, are corticosteroid-dependent, face high risk of infection, steroid side effects, and relentless disease progression. The earliest reported mortality rate in this group was as high as 68% [1], though later reports are less pessimistic: Saper et al. [2], estimate five-year survival at 42% while Schulert et al. [3], are seeing 95% survival rate in the cohort of patients followed by their group.

Despite lower mortality rates, good disease control in these patients is still hard to achieve. Given limited understanding of the underlying pathology, these patients are empirically treated with multiple immunosuppressive medications including both biologic and non-biologic DMARDs, while remaining on daily steroids. Further, there is a suspicion that the IL-1 and IL-6 inhibiting biologics may contribute to the development of the lung complications. In general, the level of immunosuppression in these patients is unsustainable long-term. Numerous infections, corticosteroid side effects and frequent admissions lead to a dramatically decreased quality of life. Marked growth retardation with complete growth cessation in some patients contribute to social isolation and depression. As a result, hematopoietic stem cell transplantation (HSCT) has emerged as a potential alternative therapeutic strategy.

The earliest report of hematopoietic stem cell transplantation (HSCT) in an SJIA patient with pulmonary disease was published in 2018, followed by a few case reports. Since 2021, however, based on the information available through patient-driven networks, the idea of HSCT has become more acceptable to both parents of SJIA patients as well as the medical teams caring for SJIA patients. Improved conditioning regimens have led to the hope that re-setting the immune system through HSCT would lead to a complete remission of the systemic JIA and associated lung disease thus abolishing the need for immunosuppressive medications and corticosteroids.

Teams with expertise in HSCT for SJIA that typically including both a rheumatologist and bone marrow transplant (BMT) specialist, were invited to participate in the NextGen 2022 session focused on HSCT in SJIA with lung disease. The parent perspective was shared by two parents of SJIA patients who underwent transplantation. Finally, Dr. Silva who had published a case series on allogeneic transplantation in JIA (which included some SJIA patients) in 2018 was invited to present the long-term follow-up data on patients from that cohort.

The goals of this NextGen session were to explore whether HSCT could be an effective strategy for patients who have become dependent on steroids and are on unsustainable levels of immunosuppression, or whose lung disease is progressing. The key questions were:

1. Since preservation of good pulmonary function is an important predictor of good outcome, can HSCT be done safely in patients with SJIA-LD whose lungs are already damaged? The degree of lung damage varies between SJIA-LD patients with many of them requiring night-time or full-time oxygen supplementation. Furthermore, some patients need tracheostomy and are ventilator dependent. Can these patients withstand HSCT?
2. The innate immune cell activation appears to be the driving force in refractory SJIA pathology suggesting that bone marrow derived cells are key players in this disease. On the other hand, one of the main risk factors for the lung disease in SJIA is strikingly high levels of serum IL-18. Although the sources of IL-18 in SJIA have not been fully defined, there is some data suggesting that it is coming not only from the bone marrow- derived cells but also from the barrier tissues such as the epithelium gut. If this were the case, would HSCT be effective at controlling the immune disease and normalizing IL-18?
3. Phenotypically, the lung disease in these patients is a distinct type of interstitial lung disease with interstitial lymphocytic infiltration and features of pulmonary alveolar proteinosis that is typically caused by dysfunction of macrophages. Can re-setting the immune system help control the lung disease? Will it stop LD progression and reverse some of the damage? Most of the previous experience with HSCT in SJIA is based on patients who did not have lung disease, so at this stage it is not clear what impact of HSCT on the lungs might be.
4. Another critical question is the optimal timing of the HSCT. Pre-HSCT organ damage, particularly pulmonary dysfunction, decreases the chances for a successful outcome of the procedure. Does moderate or severe lung damage mean that these patients are no longer good candidates for HSCT? If HSCT is indeed indicated, at what stage should it be considered? After failing multiple lines of approved medications? Should other non-approved medications be tried first, or is it preferable to proceed with HSCT before organ damage reaches a certain degree? Indeed, the rate of lung disease progression varies between patients - some do not acquire much lung damage after years of SJIA-LD diagnosis, while others are admitted to the ICU at the time of the initial discovery of lung involvement.

### Transplantation in SJIA - a brief background

#### Autologous transplantation in SJIA

Between 1999 and 2007, multiple groups in Europe reported on treatment with autologous transplantation [4, 5] for patients with severe refractory arthritis and chronic inflammation. These patient cohorts were resistant to all drugs and had become steroid dependent. Transplant outcomes were mixed. Some patients did achieve remission, but there was also significant morbidity (especially disease relapse and MAS) and mortality.

#### Allogeneic transplantation in SJIA

In 2018, Dr. Silva and colleagues published a set of case studies of Allogeneic HSCT in refractory SJIA & JIA from Europe & US [6]. Notably, those cases had been diagnosed either as RF-negative Poly JIA or SJIA. They either had unremitting arthritis and/or MAS or were steroid dependent. Dr. Silva’s review highlighted that allogeneic transplantation could be effective in JIA & SJIA and lead to disease remission, though there were some relapses.

The HSCT in SJIA-Lung Disease session was introduced by the moderator Dr. Scott Canna. Parent participants were Luciana Peixoto & Pauline Acevedo. Long-term follow-up of allogeneic HSCT cases in SJIA was presented by Dr. Juliana Silva. Case presentations were provided by Elvira Cannizzaro & Ulrike Zeilhofer, Johannes Roth & Donna Wall, Karen Onel & Susan Prockop, Pierre Quartier & Bénédicte Neven, Kyla Driest & Rolla F. Abu-Arja, and Grant Schulert & Rebecca Marsh.

### Understanding the patient experience with HSCT

Parents of two children diagnosed with SJIA-LD, who had undergone HSCT recounted their experience. They shared their child’s disease history, how the families and medical teams came to the decision to proceed with HSCT, the experience during transplantation, complications, and finally, their lives after HSCT.

#### Patient story 1

##### Parent participant: Luciana Peixoto

Mrs. Peixoto’s daughter, Beatriz, with previously normal health, was diagnosed with SJIA in September 2013 at Zurich Kinderspital. She was 11 years old at the time of diagnosis. For the next 5 years, her medical team tried several medications including multiple biologics, but could not control her flares completely and they were unable to wean her off steroids. Beatriz had one overt MAS episode and multiple episodes of sub-clinical MAS. Additionally, she had adverse reactions to biologics (rash and liver enzyme elevation) and numerous side effects from high-dose steroids prompting initial discussions about HSCT for her difficult to control disease.

In 2016, after a drop in oxygen saturation, Beatriz was diagnosed with pulmonary hypertension and lung disease. She was mostly on prednisolone at this point (as there were no further medication options available at that time). Her quality of life was poor. When her medical team brought up the idea of HSCT again, the family and the patient herself were in agreement that HSCT was the best option.

Mrs. Peixoto gained further confidence in the plan after learning that her medical team had consulted with Dr. Juliana Silva from Newcastle who was following a case series of HSCT in SJIA. Her team was planning to follow the same protocol. Additionally, her team had identified a suitable unrelated HLA matched donor, and had a backup plan of using the patient’s own stored cord blood stem cells if the allogeneic transplantation were not successful.

The transplantation performed in March 2018 went relatively well, with side effects that included hair loss, mucositis, fever, low kidney function, increased need for oxygen, morphine for pain, and blood transfusions. Beatriz was discharged home on Day + 30. About two weeks later she developed three vertebrae compression fractures due to osteoporosis and was in a lot of pain. At some point, the fever came back (MAS episode was suspected). She was admitted to the hospital for three more weeks and started to recover. Her lungs improved and over the next year, all signs of pulmonary hypertension resolved.

Four years post-transplant, Beatriz remains on Spiriva for lungs and hormone patch. She is a healthy young adult studying biochemistry and hoping to work as a researcher in drug development for rare diseases.

#### Patient story 2

##### Parent participant: Pauline Acevedo

Mrs. Acevedo’s daughter, Valentina, was initially diagnosed in 2013 with atypical Kawasaki. Next, familial hemophagocytic lymphohistiocytosis was suspected. Finally, at the age of 18 months, she was diagnosed with SJIA. For the next four years, in spite of multiple medication changes (including multiple biologics) and high-dose steroids, her disease remained poorly controlled. In 2016, the parents started noticing digital clubbing and a persistent dry cough. A year later, a bronchoscopy and lung biopsy, performed at Cincinnati Children’s Hospital, confirmed the diagnosis of Interstitial Lung Disease & Pulmonary Alveolar Proteinosis.

The biologics were stopped based on the suspicion that they were causing the lung disease (later they confirmed that her daughter carries the HLA type associated with SJIA-Lung Disease). Valentina stayed on jak-inhibitors and cyclosporine, but her physicians were unable to reduce oral steroids and she suffered from many side effects. In consultation with her medical team, the family decided to proceed with HSCT primarily due to not being able to wean steroids and uncontrolled SJIA.

She was 9 years old when her first HSCT was performed in April 2021. Although her donor was fully matched (10/10), she rejected the graft. The team started a search for another donor and the best option available was to use her 5/10 haploidentical father. This second was performed in May 2021. The complications included central line infection, mucositis, an increased oxygen requirement, an ICU stay for 3 days, and mild GVHD. She stayed in the hospital for almost 4 months.

Since the HSCT, she has been doing well and has been mostly off steroids (now on a small dose of hydrocortisone due to adrenal issues from long-term usage of steroids). She has grown almost 3 inches and the digital clubbing is improving. Of note, her lungs are showing some nodules which doctors think are infection-related (perhaps PJP). She remains asymptomatic, but the team is following closely and doing chest CTs every two months. The family reports that Valentina looks and feels healthy and has a much better quality of life.

### Long term follow-up from earlier study of allo-HSCT in SJIA published by Silva et al., in 2018.

#### Presenter: Juliana Silva (BMT), GOSH, United Kingdom

Dr. Silva presented a review and update of the patients from her previous publication [6] that included 16 patients with SJIA who underwent allogeneic HSCT from five European and US centers. Of the 16 patients, 5 had rheumatoid factor–negative polyarticular JIA, and 11 had SJIA refractory to standard therapy. Of the 11 SJIA patients, 5 also had a history of MAS, and 2 had failed previous autologous HSCT.

The median follow-up was 2 ½ years. Eight patients were transplanted with matched unrelated donors (MUD), 4 with matched sibling donors (MSD), and 4 with mismatched unrelated donors (mMUD). Reduced toxicity conditioning regimens were mostly used with Fludarabine, Melphalan, and Campath in 10 patients and Fludarabine, and Treosulfan in 6 patients.

Below are summary long-term results for the patients transplanted by 3 of the centers:

- The Great Ormond Street Hospital (GOSH) experience: This center transplanted 5 patients. Four of them achieved Complete Remission (CR), while 1 died due to transplant-related toxicity. This patient had a previous autologous stem cell transplant. Two of the four surviving patients relapsed post-HSCT. One patient relapsed 2-year post-transplant with macrophage activation syndrome (MAS) that was treated and responded well to corticosteroids. That patient is now 8-years post HSCT and remains in complete remission (CR). The second patient relapsed 10 years post HSCT and is now on treatment for arthritis.
- The Newcastle experience: The team reported 8 patients. Two patients achieved CR at 0.66 years (and at last follow-up, 9 years post-HSCT), and they remained in complete remission (CR). One patient achieved partial remission (PR), 3 patients relapsed (relapses were late at 1, 2, and 3 years post-HSCT), 1 patient died from transplant-related toxicity (TMA), and 1 patient had no response (after HSCT this patient was diagnosed with PRG4 mutation - Camptodactyly-arthropathy-coxa vara-pericarditis syndrome).
- The UCLH experience: The center performed HSCT for 3 patients. All achieved CR. One patient had declining chimerism 6 months post HSCT with autologous reconstitution but remains in clinical CR. One patient is only 2 months post-transplant in CR and it is too early to evaluate his long-term outcome.
- The CCHMC experience: The team from CCHMC described two patients transplanted at CCHMC, who are 13, and 8 years post-HSCT respectively. Both are doing well and remain in remission. 

In summary, 16 patients received allo-HSCT for SJIA. The median follow-up is 8.5 years. Eleven patients achieved CR, 2 PR, and 1 patient had no response (who was later found to have PRG4 mutation). There were 2 deaths due to transplant-related toxicity. Six patients relapsed (time to relapse post-transplant ranged from 1 to 10 years).

Dr. Silva noted that although these results show some promise, there are many unanswered questions. More data is needed to understand how to identify the subgroup of patients that will benefit from HSCT and to define the optimal timing for transplantation. Additionally, it is important to understand why some patients relapse after several years. Future prospective studies are needed to address these questions and to standardize transplant procedures.

### Brief reports of new cases presented at NextGen 2022 conference

Overall, 9 cases of HSCT for refractory SJIA were presented from 6 teams. Of the 9 patients, 5 had SJIA-Lung Disease, 1 case had some lung involvement with normal CT, 1 case had pneumonitis, while the other 2 cases were of refractory SJIA without lung disease. Six of the 9 patients had the HLA type (HLADRB1*15) that has been associated with SJIA-Lung Disease. A brief description of each case presented at the conference follows next.

### The Zurich experience: 3 cases of HSCT in SJIA, 2 with SJIA-Lung disease

#### Presenters: Elvira Cannizzaro (rheumatologist) & Ulrike Zeilhofer (BMT), University of Children’s Hospital Zurich, Switzerland

##### Case 1

The first patient was an 11-year-old female diagnosed with SJIA who had initially presented with fever, rash, splenomegaly, and hyperferritinemia. She was initially treated with prednisone, methotrexate and tocilizumab then switched to canakinumab, anakinra, and cyclosporin, with frequent prednisone pulses over 4.5 years. Despite higher doses of canakinumab, the patient was having recurrent MAS episodes and developed persistent inflammation with signs of chronic, interstitial lung disease. She later developed pulmonary hypertension. She remained steroid-dependent leading to significant side effects including osteoporosis, cataract, and growth stagnation. Additionally, she had an anaphylactic reaction to solumedrol IV and was switched to dexamethasone. Despite aggressive treatment with biological therapy, she had recurrent MAS and interstitial lung disease with pulmonary hypertension.

In 2018 (about 4.5 years into her disease), she received a 9/10 MUD transplant with a conditioning regimen that included Fludarabine (30 mg/m2/day from days − 8 to -3) and (Treosulfan 14 mg/m2/day from days − 5 to -3). For GVHD prophylaxis she received Campath (0.2 mg/m2/day from days − 8 to-4), cyclosporin, and mycophenolate. She remained on dexamethasone through transplant for 2 months.

The patient’s initial course was uneventful. She attained neutrophil engraftment on Day + 22 with 100% donor chimerism but later developed severe back pain with MRI findings of several osteoporotic vertebral bone fractures. She was treated with opiates and bisphosphonate infusions. Next, she developed CMV reactivation with secondary MAS with pancytopenia. Due to severe osteoporosis, she was treated with cyclosporin to avoid prednisone. Her recovery was slow, but she achieved full remission with improved lung function with no overnight oxygen requirement and resolving pulmonary hypertension with full donor chimerism on the last follow-up (3 years after transplant, last chimerism check was in June 2019).

##### Case 2

The second patient was a 2 ½ years old male with a similar disease presentation including fever, skin rash, recurrent MAS, splenomegaly, and severe arthritis leading to immobility with interstitial lung disease (ILD) and oxygen dependency.

He received a 12/12 HLA MUD transplant with the same conditioning regimen as Case 1 above. He attained neutrophil engraftment on Day + 20 but had a more complicated initial course with severe nausea, mucositis, several episodes of neutropenic fevers, HHV6 reactivation, E-coli sepsis, and several vertebral fractures. Following his initial discharge, he developed recurrent post-transplant MAS which was treated with methylprednisolone and then switched to dexamethasone per the HLH protocol, with a slow taper and no recurrence of MAS. On the last follow-up, he was found to have mixed donor chimerism (CD14 86% and CD3 76%) but he was doing clinically well off immunosuppression with improved lung disease and quality of life.

Both Cases 1 & 2 were of patients with refractory systemic JIA who were steroid-dependent leading to the development of significant side effects including osteoporosis, cataract, and growth stagnation. Despite aggressive treatment with biological therapy, they both had recurrent MAS and interstitial lung disease. Although both patients had a complicated transplant course both patients have achieved full remission and are doing well post-transplant.

##### Case 7

The most recent case from Zurich is of a 3-year-old girl transplanted in November 2020. She had multiple MAS episodes, polyarthritis, was steroid dependent (with severe side effects). She also had allergic reactions to several medications.

She was transplanted with the same conditioning regimen as the first 2 patients from Zurich: Treosulfan /Fludarabine/ Campath. Her match was 10/10 matched unrelated donor and the transplantation course was uneventful. However, all donor cell lines (CD14, CD 15, and CD3) dropped around 3–4 months after transplantation. At first, the arthritis was still controlled, but once steroids were stopped, she relapsed with her JIA that was again difficult to control.

The team decided to proceed with a second HSCT which was performed in November 2021. She had another 10/10 matched unrelated donor. This time, the team decided to use a more intense conditioning regimen: Busulfan/Cyclophosphamide/ATG. The transplantation course was initially uncomplicated, but in January 2022, she had a generalized seizure and was diagnosed with EBV encephalitis. Although clinically she appeared well, there were concerning findings in the MRI and CSF. She was treated with Valcyte, Rituximab, and one dose of CD45 + RO cells with a good response. Currently, chimerism is at 100% percent for all cell lines. Steroids were stopped at the end of January 2022, but it remains too early to assess if the second HSCT will lead to long disease remission.

### The Paris experience: 1 case of Haploidentical HSCT in SJIA

#### Presenters: Pierre Quartier (Rheumatology) & Bénédicte Neven (BMT), Necker-Enfants-Malades University Hospital, Paris, France

##### Case 3

The team presented 2 patients, a case of HSCT for refractory SJIA performed 5 years ago [7] as well as a second HSCT for a refractory SJIA patient, who was transplanted very recently.

This center had previous experience with autologous transplantation in a few patients with severe Still’s Disease, but the patients presented with numerous complications after transplantation and continued to have flares.

Their first allogeneic transplantation in 2017, was a female SJIA patient, 3.7 years old who fulfilled the eligibility criteria for HSCT. She had a severe systemic disease but did not have interstitial lung disease. She had proven refractory to both IL-1 and IL-6 blockers, as well as to thalidomide, methotrexate, and infliximab.

The mother was the donor (although she had vitiligo), as there was no other donor available. The haploidentical bone marrow transplantation was performed with a conditioning regimen of busulfan, fludarabine, campath, and post-transplant cyclophosphamide. She achieved full donor chimerism, but several complications occurred in the post-transplantation course: acute skin and gastrointestinal GVHD (treated with steroid therapy), infections and thrombocytopenia treated with intravenous immunoglobulins. At last follow-up, apart from vitiligo and asymptomatic hypothyroidism, the patient achieved full remission from arthritis and systemic disease with 100% chimerism.

The other SJIA patient was transplanted 2 months ago. She is a 3-year-old with SJIA. She has had recurrent MAS and her disease has proven refractory to IL-1 blockers, tocilizumab, and baricitinib. After a severe SJIA flare without MAS, sirolimus was introduced. She has been on emapalumab for MAS and high-dose steroids. The patient had very high IL-18 levels consistent with the phenotype of young children who do not respond to biologics and are at risk of lung disease.

### The Columbus experience: 1 case of HSCT in SJIA with lung involvement

#### Presenters: Kyla Driest (Rheumatology) & Rolla F. Abu-Arja (BMT), Nationwide Children’s Hospital, Columbus, United States

##### Case 4

The team at Nationwide Children’s Hospital presented a 16-year-old female patient who initially presented with fever, rash, and arthritis [8]. The patient was initially diagnosed with Celiac disease and then, SJIA. She was started on prednisone and etanercept. Due to continued arthritis, etanercept was replaced with tocilizumab followed by adalimumab.

The patient had multiple admissions for fevers that were treated as infections with antibiotics while holding biologic therapy. On her 4th admission (5 months after the initial diagnosis) she developed overt MAS and her treatment regimen was changed to the combination of cyclosporin and canakinumab.

Her care was transferred to Nationwide Children’s at this point. Given the continued disease activity and side effects, cyclosporin was replaced with leflunomide and hydroxychloroquine. The Canakinumab dose was increased to 300 mg every 4 weeks and then to 500 mg every 4 weeks due to smoldering MAS. She was then admitted for a severe MAS flare with limited response to high-dose solumedrol (1-gram IV Q daily). Next, her treatment was changed to anakinra, dexamethasone, IVIG, and tacrolimus. During this time, she started showing signs of dyspnea on exertion. Pulmonary function testing revealed low DLCO and mild obstruction with normal chest CT and echocardiogram.

At the age of 20, in 2018, she received an allogeneic bone marrow transplant from a 30-year-old male 10/10 HLA MUD using a reduced-intensity conditioning regimen (Intermediate Alemtuzumab 0.3 mg/kg/dose SQ for 3 days (days − 14 to -12) Fludarabine 30 mg/m2/dose for 5 days (days − 8 to -4) and Melphalan 70 mg/m2/dose for 2 doses (days − 2 and − 1)). GVHD prophylaxis consisted of tacrolimus and methylprednisolone while she continued anakinra daily until engraftment and discontinued hydroxychloroquine before the start of the conditioning regimen. She achieved engraftment on Day + 9 and was discharged on Day + 14. Her peripheral blood chimerism was 99–100% donor in all cell lines. Complications included Grade 1 skin GVHD treated with topical corticosteroid therapy. She also developed EBV reactivation on Day 100 for which she received Rituxan which caused B cell aplasia lasting for 1 year. She was weaned off tacrolimus at 6 months post-transplant and prednisone at 18 months due to secondary adrenal insufficiency. Due to prolonged prednisone use, she had slow immune reconstitution and remained on replacement IV immunoglobulin for 2 years post-transplant.

Based on the last follow-up, the patient is now 3 ½ years post HSCT with complete resolution of arthritis, improvement in lung function, and DLCO. She is off immunosuppression with full immune reconstitution. Her Celiac disease has resolved.

### The Emory-Atlanta experience: 1 case of HSCT in SJIA

#### Presenters: Sampath Prahalad (Rheumatology) and Shanmuganathan Chandrakasan (BMT), CHOA, Emory-Atlanta, United States)

##### Case 5

This case from Emory was that of a 19 year old African-American female referred for HSCT due to refractory SJIA, complicated by multiple MAS flares, and bacterial infections (primarily cutaneous abscesses). Her disease started at the age of 14 years and quickly evolved to a steroid-dependent SJIA state despite treatment with anakinra, tocilizumab, methotrexate, and cyclosporin A.

During the year preceding the HSCT, she had five MAS flares, with two of them requiring an ICU admission. Her course was also complicated by CMV viremia and pneumonitis treated with ganciclovir. Many of her MAS/SJIA flares were also associated with muscle enzyme elevation. An immunologic workup revealed very high IL-18 levels (> 200,000). In addition, a workup for HLH revealed a single allele mutation in STXBP2 and LYST, but her CD 107 degranulation assay (a test for the functional integrity of the cytotoxic T cell degranulation (CTL) pathway) was normal. Based on that, primary HLH phenotype was felt unlikely.

Due to the refractory disease course, the team elected to proceed to allogeneic HSCT as a potentially curative option. At the age of 19 years, the patient underwent 9/10 mismatched unrelated PBSC graft HCT following Campath/ Flu/ Mel/ Thiotepa conditioning. Peri-conditioning (despite Campath and fludarabine), she had a disease flare. Due to this flare during conditioning, she was re-started on anakinra. She achieved engraftment on Day 11 with 100% donor cells in T and myeloid compartment. Her IL-18 levels improved from > 200,000 to 903 two months post HSCT and remained below 2000 thereafter.

Her disease course was complicated by acute Grade III GVHD of the GI/ Skin, posterior reversible encephalopathy syndrome, and TA-TMA (Transplant-associated thrombotic microangiopathy) leading to worsening renal function requiring hemodialysis and later CVVH. Her aGVHD was managed with pulse steroids, infliximab, and extra corporeal photopheresis with a partial response and continuous steroid-dependent state. She also developed CMV pneumonitis and viremia. Her TA-TMA was managed with eculizumab. Despite aggressive support, she had progressive cardiorespiratory worsening and died on Day + 133. Postmortem evaluation revealed extensive thrombotic microangiopathy with diffuse bilateral kidney necrosis with microthrombi and diffuse alveolar damage with hyaline membrane, diffuse pulmonary hemorrhage, and numerous thrombi of different stages and small and large intestine with focal ischemic necrosis with microthrombi. Based on the autopsy, the death was primarily caused by refractory TA-TMA.

### The Toronto/Ottawa experience: 1 case of HSCT in SJIA-Lung Disease

#### Presenters: Johannes Roth (Rheumatology), University of Ottawa & Donna Wall (BMT), University of Toronto, Canada

##### Case 6

The team from Canada presented a patient who received an allo-HSCT in 2020. It was a female who initially presented at the age of 3.5 years with high fevers, rash, and arthritis followed by pancytopenia, high ferritin leading the diagnosis of MAS. She was initially treated with a combination of prednisone, IVIG, and anakinra. Cyclosporine was added with no response. Anakinra was then replaced with tocilizumab, but she developed an adverse reaction to this medication, and was then switched to high dose canakinumab. Genetic panel testing was negative with whole-exome sequencing showing a variant of unknown significance in the NLRC4 gene. This variant was also present in the mother and was ultimately deemed as likely not pathogenic.

About 2 years into her disease course, she developed clubbing and lung disease. A lung biopsy confirmed the distinct phenotype associated with pulmonary alveolar proteinosis typically seen in patients with SJIA-Lung Disease. Initially, the lung disease was stable, but a year later she had another episode of severe MAS that triggered further progression of the lung disease and she became oxygen dependent. The patient participated in a trial of an anti-IL-18 agent but dropped out in the double-blind phase of the trial and it is still unknown whether she received the drug or placebo. She was also treated with high doses of tofacitinib and emapalumab, with no clear response.

The decision to proceed with HSCT was not an easy one as she was on supplemental 3–4 L of oxygen and lungs were very inflamed. It involved the family, multiple medical teams from other hospitals, as well as experts in bioethics. There was no active infection on pre-transplant bronchoscopy. The team optimized immunosuppression, and decided to continue emapalumab, steroids, and anakinra though the transplant procedure as part of graft versus host prophylaxis, as well as to control the underlying inflammatory process.

She received a 10/10 HLA MUD peripheral stem cell transplant using a reduced toxicity conditioning regimen. Pretransplant immunotherapy (emapalumab, prednisone, and anakinra) was continued through the early transplant period.

Her transplant course was complicated with significant worsening of pulmonary function requiring temporary ventilatory support and dialysis at time of engraftment. Although her transplant recovery was slow, she showed fast resolution of arthritis and was able to stop anakinra and emapalumab following engraftment.

At 3 months post-transplant, she developed features of acute and chronic GVHD primarily involving the skin but had a quick response to ruxolitinib.

The patient is now 16 months post-transplant with full donor chimerism, no clinical GVHD, and mild thrombocytopenia. Her respiratory function is improving based on imaging, resolving clubbing, and decreased oxygen support.

**Figure:**
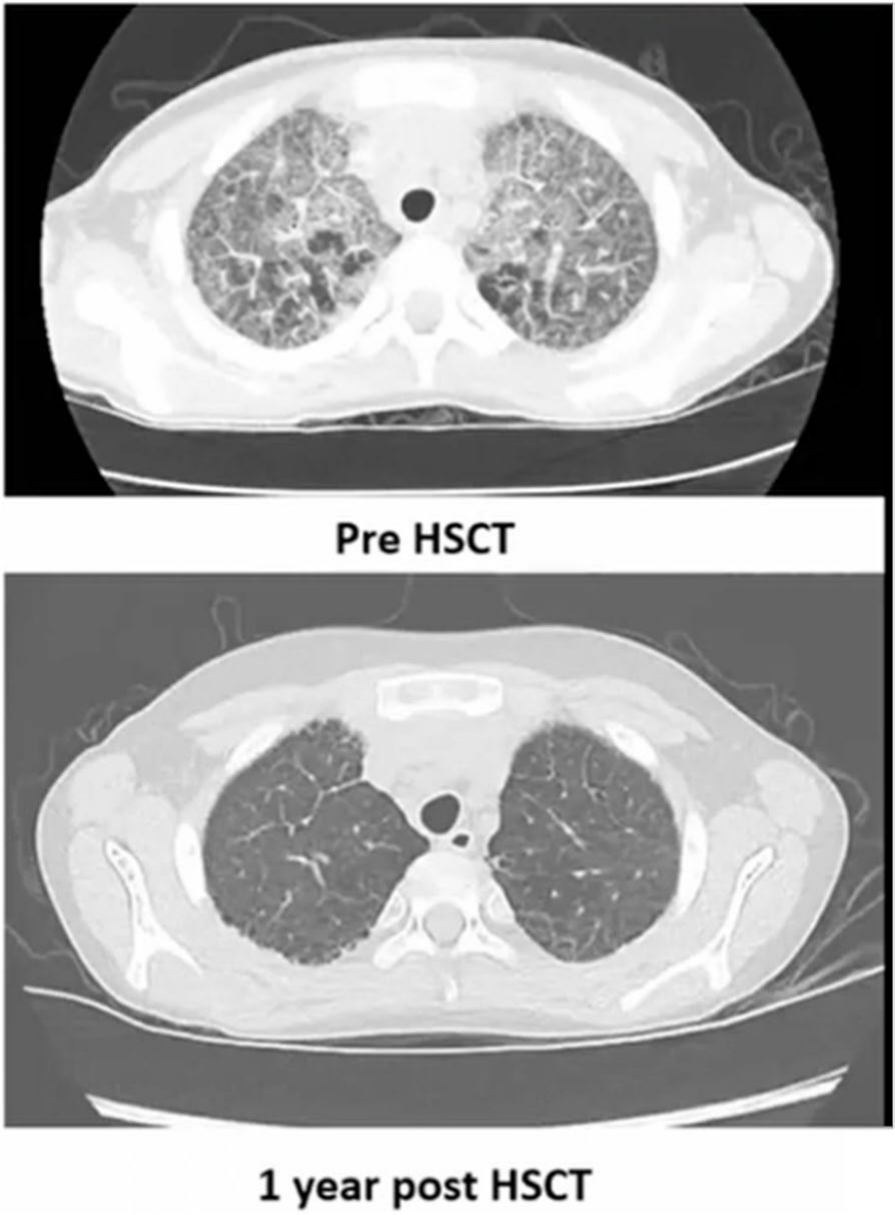
Case 6: Chest CT scan for lungs: Pre HSCT and 1-year post-HSCT

### The Cincinnati experience: 1 case of HSCT in SJIA-Lung Disease

#### Presenters: Grant Schulert (Rheumatology) & Rebecca Marsh (BMT), Cincinnati Children’s hospital, United States

##### Case 8

The female patient who, in 2013 at the age of 10 months presented with rash and fever and was initially diagnosed with Kawasaki disease but failed to improve despite treatment with IV immunoglobulins. At the age of 18 months, she developed MAS followed by arthritis and was then diagnosed with Systemic JIA. The patient was initially treated with prednisone and cyclosporine but was unable to wean off prednisone. She was then started on tocilizumab, but had an infusion reaction, and was then switched to canakinumab. She had an excellent response initially but missed multiple doses due to recurrent infections and pneumonia. She developed chronic cough and clubbing with abnormal lung findings on the chest CT scan. She continued to have recurrent MAS typically triggered by infections. A lung biopsy was performed and confirmed the diagnosis of SJIA-LD with PAP-like features.

She remained resistant to biological therapy with worsening lung disease with overnight hypoxia, recurrent MAS, chronic steroid dependence, and drug toxicity including growth failure, hypertension, and infections.

She received an allogeneic HSC transplant from a 10/10 HLA MUD using a reduced-intensity conditioning regimen of Alemtuzumab, Fludarabine, Melphalan, and thiotepa. The graft was TCR a/b CD19 depleted and there was no GVHD prophylaxis. The patient did well Initially and achieved neutrophil engraftment on Day + 13 with 100% donor chimerism, however, she developed acute secondary graft rejection on Day + 32. She received a second transplant 6 weeks following the first transplant using a parental haploidentical donor, with ATG, fludarabine with post-transplant cyclophosphamide, cyclosporin, and mycophenolate for GVHD prophylaxis. The patient did well with count therapy and 97% donor chimerism. Transplant complications included Grade 1 Stage 1 skin GVHD that responded well to steroids. The patient is now 7 months post-transplant and is doing well off immune suppression without major complications and with no systemic inflammation. There has been improvement in clubbing and cushingoid features.

**Figure:**
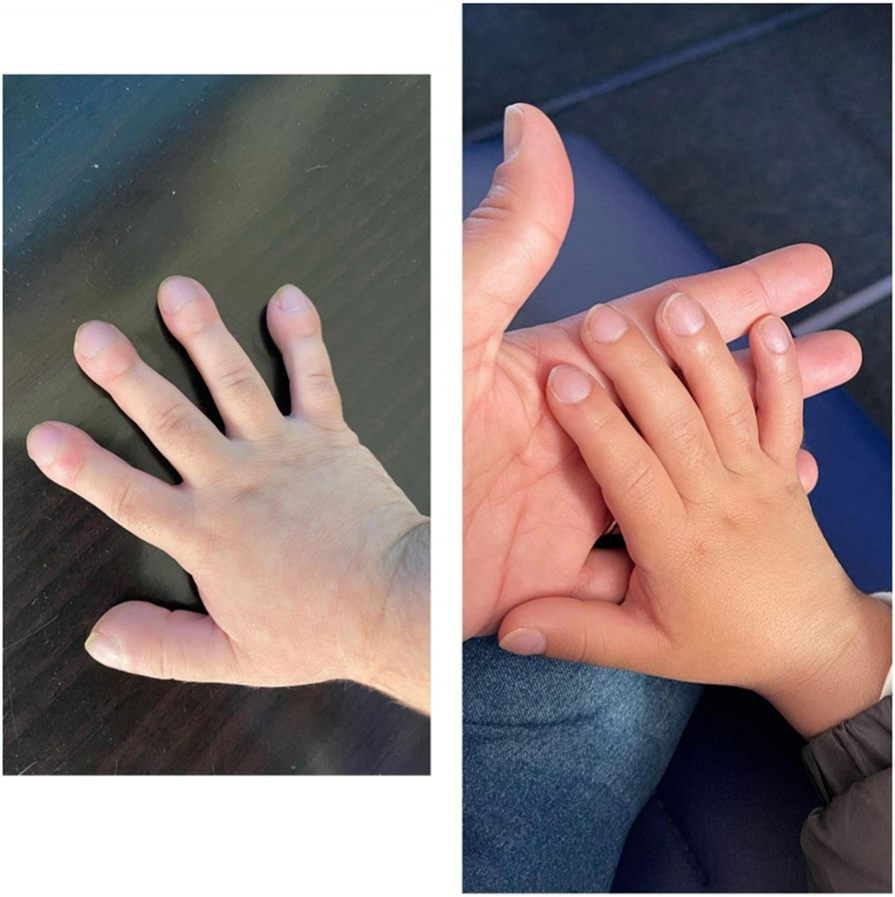
Case 8: Clubbing improvement: before and after HSCT (approximately 9 months)

### The New York experience: 1 case of HSCT in SJIA-Lung Disease

#### Presenters: Karen Onel (Rheumatology) & Susan Prockop (BMT), HSS/MSKCC/NYH-WC, New York, United States

##### Case 9

An 18-month-old male initially presented with fever, rash, and multiple-joint arthritis. For the next few years, his disease proved refractory to many medications, and he remained on high-dose steroids. At the age of 5 years, he was referred to MSKCC for consideration for HSCT. He was steroid dependent, refractory to IL-1 and IL-6 inhibitors (anakinra, tocilizumab, & canakinumab), mycophenolate, rilonacept, cyclosporin, and tofacitinib. His course was complicated by recurrent MAS, systemic hypertrophic osteoarthropathy, and interstitial lung disease with clubbing. Lung biopsy showed diffuse lymphocytic infiltration with alveolar proteinosis.

At the time of referral, he was on daily prednisone dose of 1.2 mg/kg/day and monthly pulses of methylprednisolone 500 mg and cyclophosphamide. On genetic testing, he was found to have the HLADRB1*15, and candidate gene mutation (NLK). His lung disease continued to progress with evolving tachypnea and interstitial lung disease with cystic changes that led to the decision to move forward with allo-HSCT.

His pre-transplant evaluation identified low-level CMV on bronchoalveolar lavage (BAL), for which he received induction ganciclovir prior to starting conditioning for HSCT. The patient received a haploidentical HSCT from his 7/10 mother using a conditioning regimen of Busulfan (PK 60 mg*h/L) Fludarabine, ATG, and Rituximab, with post-transplant cyclophosphamide on Days + 3 and + 4, tacrolimus, and MMF for GVHD prophylaxis.

His transplant course was complicated with persistent fevers for the first 2 weeks and at the time of engraftment on Day + 21. Several weeks after engraftment he developed increasing respiratory distress with worsening of parenchymal changes by chest CT. A repeat BAL was negative for infections except for low-level CMV. His inflammatory markers: CRP, Ferritin, and IL-6 levels were increased. He required admission to the pediatric intensive care and was placed on a high-flow nasal cannula. He received pulsed prednisone 1 mg/kg BID, resumed MMF and ultimately was discharged on overnight high-flow support.

The patient is now 8 months post-haplo-HSCT with full donor chimerism in all lineages. He remains on tacrolimus and MMF and weaning prednisone (at the time of the meeting on 0.2 mg/kg/day). His immune reconstitution is slow due to prolonged prednisone use. The patient remains on aggressive pulmonary toileting but is no longer on high-flow nasal cannula. The chest CT showed marked improvement of parenchymal changes.

#### Summary of discussions

The discussion, moderated by Dr. Canna, was wide-ranging, touching on many aspects of the cases presented. The main themes are summarized below.

 Will the most refractory SJIA patients benefit from HSCT? First, Dr. Canna raised the question of whether there was a subset of patients with refractory systemic JIA, who have failed all available medications, who could potentially benefit from HSCT. How can we identify those patients early? The refractory SJIA patients presented at NextGen were dependent on high doses of corticosteroids despite multiple immunosuppressive medications. While this population had significant peri-HSCT complications, most have achieved SJIA remission or experienced a decreased burden of SJIA, and improved lung function. The follow-up, however, has not been long enough to define the long-term outcomes.

Dr. Driest commented that rheumatologists, being aware of possible complications of HSCT, tend to keep looking for the next medicine with the hope that they will find the right one at some point. Given the number of drugs available as well as their possible combinations, this path can be very long. For their own patient, the team had changed medications multiple times and ultimately decided that it was time for a transplant rather than trying another combination.

Dr. Onel recounted that initially, their team was not in favor of HSCT. However, as the lung disease was rapidly progressing, and lung transplantation was proposed as the next step - their thinking changed. Their concern was that poorly controlled underlying SJIA would reduce chances for successful lung transplantation. Additionally, there was a concern that the inflammatory process can reoccur in the transplanted lung leading to new lung damage. A better approach might be to arrest the child’s lung disease with the ultimate immune ablation provided by HSCT.

 What is the optimal timing for transplants? / How to transplant before it’s too late, especially for lung patients? Next, Dr. Canna raised a question about the ideal timing to perform HSCT. Is it after patients have failed the approved biologic and non-biologic DMARDs or after trying other available immunosuppressive medications? The cases presented at NextGen varied in terms of SJIA duration at the time of HSCT (from 1.9 to 8.2 years) with different degree of organ damage and toxicities from treatment. For one of the patients, the transplant was such a risky procedure, that the final decision was made with the involvement of the bioethics team. Is there a way to identify these patients earlier in the course of the disease?

A parent of an SJIA-LD patient raised the question about the clinical parameters that would make a patient no longer eligible for HSCT. The consensus was that the fewer comorbidities would lead to fewer complications during HSCT. Conversely, a patient who spends years on corticosteroids and other immunosuppressants with frequent infections would be at higher risk for mortality, infections, and other side effects associated with HSCT.

Additionally, physicians agreed that although decreased pulmonary and cardiac function was a major concern, it was still possible to go through a transplant even if the patient was tracheostomy-dependent and on oxygen supplementation (with reasonable ventilator settings). In such cases, the final decision should be made by a multidisciplinary team based on very thorough evaluation.

Dr. Wall added that the transplant team must be prepared for numerous complications including ICU stays, especially if the patient had lung involvement. She added that for lung function, it is difficult to determine the exact cut-off point for HSCT eligibility when the lung is the target organ. Nevertheless, it is important to thoroughly evaluate lung function even if the imaging tests show severe lung inflammation. Dr. Grom added that SJIA-Lung patients often might be doing well and have preserved lung function despite impressive changes on high resolution chest CT.

One additional consideration is specific to patients with severe growth delays (even growth failure), who are unable to get off steroids. For such patients, there is a need for a window of time after transplantation to catch up with growth before their growth plates close. As an example, for the patient from Cincinnati who had been on corticosteroids continuously for 9 years prior to the procedure, it took 18 months to complete her steroid taper post-BMT. She started growing at 8 months after transplant.

 Need for early referrals to the BMT team. The BMT specialists emphasized the importance of early referrals of refractory patients to their teams, so they can stay involved with the patient and assess donor options.

Dr. Schulert from CCHMC shared that they were now referring any SJIA patients with a refractory course, who had failed both IL-1 and IL-6 blockade as well as jak-inhibition, to the BMT team.

 Plan for Retrospective Study and Need for Guidelines. Dr. Juliana Silva proposed starting a retrospective study with long-term follow-up of the cohort of transplanted SJIA-LD patients. She invited all the teams present to contribute to this effort that should include monitoring the rates of relapses and chimerism. She also emphasized the need for prospective studies of patients undergoing different standardized transplant procedures. Thus initially, the team used Flu/Melph/Campath as conditioning, but more recently they have changed the regimen to Fludarabine/Biosulphan with the hope that they would achieve better chimerism.

Dr. Abinun added that the existing European governmental HSCT guidelines were very conservative. He also emphasized the importance of publishing each case study so we could learn from these patients and improve these guidelines.

 Is this group of patients too heterogeneous to draw conclusions from? Session participants had varied opinions about the heterogeneity of the refractory SJIA patients. Dr. Onel pointed out that there is a larger group of refractory SJIA patients who do not necessarily have lung disease but require multiple immunosuppressive drugs and cannot wean off steroids. She felt that refractory SJIA patients with parenchymal lung disease that have PAP features were a distinct and more homogenous group. Dr. Canna and Dr. De Benedetti thought that the SJIA patients with recurrent MAS associated with any organ involvement were on the same disease spectrum, even if the specific organs involved were not the same. In contrast, Dr. Wall felt that the presented cohort of patients was still very heterogeneous with different mechanisms driving the disease. These different perspectives make it it difficult to come up with specific guidelines.

 Conditioning Regimen and Chimerism. The ideal conditioning regimen for HSCT for patients with SJIA has not been identified. Specialists from two of the centers (Newcastle & Kinderspital) observed that the reduced intensity conditioning regimen utilizing treosulfan/fludarabine might not be sufficient to ensure durable complete donor chimerism. This also came up in the long-term follow-up data presented by Dr. Silva where several of the patients relapsed and others had low chimerism. However, Dr. Silva pointed out that the relapse was not highly correlated with the drop in chimerism.

 Need to control inflammation before HSCT and medications to be continued through transplant? Different teams had different opinions about the need for the continuation of the SJIA medications through the transplantation procedure. The team from Canada emphasized the need for a full control of inflammation prior to the transplant and GVHD prophylaxis. Their patient was on anakinra and emapalumab prior to the transplant and continued these medications until engraftment. The patient from CCHMC was on emapalumab before her second transplant.

The NYC team decided to keep the patient on PO prednisone (1.2 mg/kg daily), monthly pulses of IV steroids, and cyclophosphamide during the time period leading up to the transplant, but they tried to minimize immunosuppression at the time of the graft infusion. Minimizing immune suppression in the context of haploidentical-HSCT with post-transplant cyclophosphamide is critical to the success of this approach.

Dr. Driest described their struggle with getting the dose of steroid right before the transplant for their patient who had been steroid dependent during the entire course of SJIA. They kept the patient on 20 mg of steroids as this was the dose that had previously controlled her disease. At any lower dose, she was felt to be in danger of flaring. Their patient also continued anakinra through the transplantation.

Dr. Quartier described that the French team tapered the dose of steroids during the pre-transplant conditioning regimen. They stopped the immunosuppressive treatments before HSCT, with the exception of emapalumab that was continued several weeks after HSCT in the second French patient.

 Risk Post-Transplant MAS. Two of the teams reported MAS post-transplant (Case 1 and Case 2). Case 1 had just 1 episode, treated with cyclosporin. Case 2 had multiple episodes that were resolved with steroids pulses and ultimately, dexamethasone pulse. One of the cases (Case 5) reported a flare during conditioning regimen, pre HSCT, and she was re-started on anakinra. The team for Case 9 reported a post-transplant episode of lung disease where it was unclear if it was MAS or another reason. That episode did resolve after treatment with steroids. Additionally, two patients (Case 6 & Case 8) who both had history of MAS were treated with emapalumab right before transplant and a few doses post-transplant.

 Improvement in Lung Disease post-transplant. The Toronto/Ottawa team had seen significant improvement in the chest CT a year after the transplant. The patient, however, remained on ruxolitinib post-transplant, and there was a question as to whether the improvement in the lung disease was due to HSCT or to the ruxolitinib. Indeed, ruxolitinib has been effective for lung disease in primary immunodeficiencies like CT04. This also raised a second question whether ruxolitinib should be used for lung disease in SJIA before transplant in general. Dr. Wall mentioned that their patient had failed a different JAK-inhibitor prior to the transplant. Currently, the team is happy with the lung response and does not want to discontinue ruxolitinib. The fascinating part for them was that they had started sirolimus for an atypical rash for GVHD, and the rash exploded. They stopped sirolimus and went to ruxolitinib and the rash melted away. Overall, the team felt humbled with the results of this challenging transplant. They emphasized the importance of controlling the inflammation pre-transplant and continuing the medications as part of GVHD prophylaxis. Dr. Roth added that he had been surprised by how reversible the advanced lung disease was in their patient. Even if some of this improvement could be attributed to the ruxolitinib, his team felt that most of the benefit was coming from resetting the immune system via HSCT. He also mentioned that they are increasing the use of ruxolitinib in patients for MAS.

 HLA Type. Dr. Canna also raised the question about the role of HLA-DRB1*15 which has recently been identified as a risk factor for the lung disease in SJIA patients [2]. He was wondering whether the HLA type should be taken into consideration while looking for a donor. Dr. Prockop replied that typical donor HLA matching done for HSCT is more important for transplant outcome than considering the SJIA-LD HLA type as a way to screen donors.

 Long term morbidity. Dr. Nigrovic raised the issue of long-term morbidity after transplant, particularly secondary malignancies. The BMT specialists pointed out that they did not see any such issues in their cases so far, though it is too soon to tell with this current cohort. Dr. Marsh thought that there was a risk of secondary malignancies, while Dr. Wall pointed out that so far, there were no reports of second malignancies for pediatric patients with inflammatory disorders undergoing HSCT.

 Autologous Transplant. Dr. Canna next raised the question of whether autologous transplantation should be considered for these patients. Dr. Silva pointed out that autologous transplants were performed in SJIA in the past, but there were several relapses, and/or patients did not go into remission. Dr. Wall added that a reset of the immune system might be sufficient for adult patients, but not for pediatric patients (where genetic defects are more likely to be part of the pathology compared with adults), and therefore they need an allo-HSCT.

Dr. Abinun added that with autologous transplantation cases, MAS was a major issue in these patients, and that reducing inflammation (pre-transplant) was the way to solve this problem. He mentioned that the Dutch group was closely studying the immune reconstitution following autologous transplantation and hopefully this research would provide more answers in the future.

 Control of inflammation before HSCT, Chimerism and SJIA remission. The importance of a good control of inflammatory activity prior to HSCT was again brought up by several BMT specialists. Dr. Abinun commented that most issues he sees in these patients during transplantation are inflammation-related, and the same issues can also have an impact on the post-HSCT course including chimerism. He also finds is disheartening that even full donor chimerism in SJIA patients after HSCT, does not mean that the disease is cured. Perhaps, this suggests a role for non-hematopoietic cells in the development of SJIA. He discussed these issues in more detail in his recent publication [9].

 Insurance issues. All teams reported that they did not have insurance issues in HSCT getting approved. Dr. Marsh mentioned that for other similar patients who have an inborn error of immunity and no genetic diagnosis, they usually send a letter to the insurance company as part of the transplant package. It might take a follow-up conversation with someone at the insurance company but typically it’s not a barrier.

 Conditioning for refractory SJIA associated with liver disease. One of the parents asked about HSCT in patients with SJIA and liver involvement. Several BMT experts agreed that for patients with liver disease, there is a need to adjust the conditioning agents used.

##### Summary and future directions

In summary, the entire group of the participants agreed that there is a subset of patients with refractory systemic JIA, who have failed all available medications and are likely to benefit from HSCT. The increasing experience with HSCT in SJIA, suggests that despite the complicated post-HSCT course, short-term, the transplanted patients either achieved SJIA remission (or reduced burden of disease) with improved lung function. Longer follow-up, however, is needed to better define the long-term outcomes.

The optimal timing for the procedure still needs to be determined, but the emerging consensus is that it should occur earlier before significant organ damage is accumulated. The group unanimously agreed that BMT teams should be consulted early, and both Rheumatology and BMT specialists should work together to determine when HSCT is appropriate. Increased pre-HSCT organ damage, lungs in particular, decreases the chances for a successful outcome of the procedure. However, since lungs are the target organ in SJIA-LD, patients with moderate or even severe lung damage still may be eligible for the procedure.

Although reduced intensity conditioning regimens have improved the outcome of HSCT in general, there is a remote concern that such regimens may increase the risk of SJIA relapse after the transplantation. Long term registries should help address this question.

The entire group agreed that a good control of inflammatory SJIA activity prior to HSCT markedly increases chances to achieve a good outcome, and therefore, it is reasonable to continue SJIA/MAS medications until transplantation. The need to continue these medications (emapalumab and Jak-inhibitors in particular) after the procedure still needs to be determined.

## Data Availability

All the data discussed during the meeting have now been published and appropriately referenced at the end of the manuscript.

## Abbreviations

ATG: Anti Thymocyte Globulin
BMT: Bone marrow transplant
PO: By mouth, orally
CSF: Cerebrospinal fluid
CR: Complete Remission
CT: Computerized tomography
CMV: Cytomegalovirus
CTL: Cytotoxic T cells
DLCO: Diffusing capacity of the lungs for carbon monoxide
DMARDS: Disease-modifying anti-rheumatic drug
EBV: Epstein-Barr Virus
GI: Gastrointestinal
GVHD: Graft-versus-host disease
HSCT: Haematopoietic Stem Cell Transplantation
HLA: Human Leukocyte Antigen
ICU: Intensive care unit
IL: Interleukin
IV: Intravenous
JIA: Juvenile Idiopathic Arthritis
LD: Lung disease
MAS: Macrophage Activation Syndrome
MRI: Magnetic resonance imaging
MSD: Matched sibling donors
MUD: Matched unrelated donors
mMUD: Mismatched unrelated donors
MMF: Mycophenolate mofetil
PR: Partial remission
PJP: Pneumocystis jiroveci pneumonia
PAP: Pulmonary alveolar proteinosis
SJIA: Systemic Juvenile Idiopathic Arthritis
TA-TMA: Transplant-associated thrombotic microangiopathy
TMA: Transplant-related toxicity
